# S1 Constrains S4 in the Voltage Sensor Domain of Kv7.1 K^+^ Channels

**DOI:** 10.1371/journal.pone.0001935

**Published:** 2008-04-09

**Authors:** Yoni Haitin, Ilanit Yisharel, Eti Malka, Liora Shamgar, Hella Schottelndreier, Asher Peretz, Yoav Paas, Bernard Attali

**Affiliations:** 1 Department of Physiology and Pharmacology, Sackler Medical School, Tel Aviv University, Tel Aviv, Israel; 2 The Mina and Everard Goodman Faculty of Life Sciences Bar-Ilan University, Ramat-Gan, Israel; Emory University, United States of America

## Abstract

Voltage-gated K^+^ channels comprise a central pore enclosed by four voltage-sensing domains (VSDs). While movement of the S4 helix is known to couple to channel gate opening and closing, the nature of S4 motion is unclear. Here, we substituted S4 residues of Kv7.1 channels by cysteine and recorded whole-cell mutant channel currents in *Xenopus* oocytes using the two-electrode voltage-clamp technique. In the closed state, disulfide and metal bridges constrain residue S225 (S4) nearby C136 (S1) within the same VSD. In the open state, two neighboring I227 (S4) are constrained at proximity while residue R228 (S4) is confined close to C136 (S1) of an adjacent VSD. Structural modeling predicts that in the closed to open transition, an axial rotation (∼190°) and outward translation of S4 (∼12 Å) is accompanied by VSD rocking. This large sensor motion changes the intra-VSD S1–S4 interaction to an inter-VSD S1–S4 interaction. These constraints provide a ground for cooperative subunit interactions and suggest a key role of the S1 segment in steering S4 motion during Kv7.1 gating.

## Introduction

Voltage-gated potassium channels (Kv) are key regulators of cellular excitability by shaping action potentials, tuning neuronal firing patterns, synaptic integration and neurotransmitter release [1]. Kv channels comprise four subunits arranged symmetrically around a central ion-conducting pore. Each subunit consists of six transmembrane segments, including an S5–S6 region encompassing the aqueous pore and a peripheral S1–S4 voltage sensor domain (VSD). A large body of evidence indicates that the first four arginine residues in S4 account for most of the 12–13 electronic charges per channel that are translocated across the membrane's electric field [2], [3]. Although it is well accepted that the movement of the voltage-sensing S4 helix is tightly coupled to opening and closing of the cytoplasmic S6 channel gate, the nature of S4 motion is uncertain. Specifically, the topology of the VSD in the channel closed state and the magnitude of the S4 movement following depolarization remain controversial. So far, three main models of VSD motion have been proposed: (i) the transporter model, in which S4 moves only a small distance (2–3 Å), but through a focused and mobile electric field within an aqueous crevice whose accessibility changes during gating [4]; (ii) the helical screw model, in which the S4 helix rotates clockwise and translates outward (∼13 Å) along its axis to move the gating charges across the membrane electric field [5], [6]; (iii) the paddle model where the sensing unit (S4-S3b) undergoes a large transverse movement (∼15–20 Å) across the membrane and in which the S4 arginines are mostly exposed to lipids [7].

While great efforts have been dedicated to elucidate the nature of the VSD motion using *Shaker* channels as a general model, virtually no study has addressed this key issue in Kv7.1 channels [8]. This question is particularly pertinent in light of the pathophysiological importance of cardiac Kv7.1 (KCNQ1) channels and their unusual slow gating kinetics arising from their native co-assembly with the KCNE1 β subunits. In this work, we substituted residues along the S4 N-terminus and the short S3–S4 linker with cysteines and studied their propensity to form metal or disulfide bridges, using Cd^2+^ or copper-phenanthroline (Cu-Phen), respectively. Experimental data and structural modeling constrain the Kv7.1 closed state to an intra-VSD S1–S4 interaction and, upon depolarization to an inter-VSD S1–S4 interaction. In the accompanying paper, we show that KCNE1 interferes with the inter-VSD S1–S4 interaction and thereby modulates Kv7.1 voltage sensor properties.

## Results

**Figure 1:**
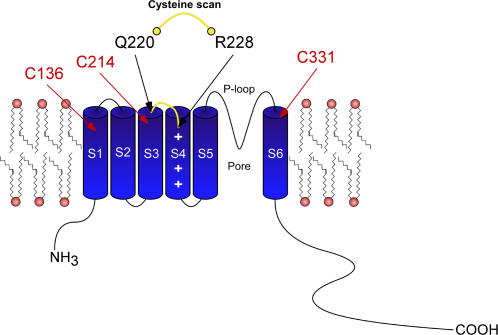
Schematic cartoon showing the S3–S4 linker and the S4 N-terminal region of the Kv7.1 channel where the cysteine scan was performed. In red labels are shown the three endogenous cysteines accessible from the external solution, which could potentially form metal or disulfide bridges (C136, C214 and C331, located in the S1, S3 and S6 transmembrane segments, respectively).

**Figure 2:**
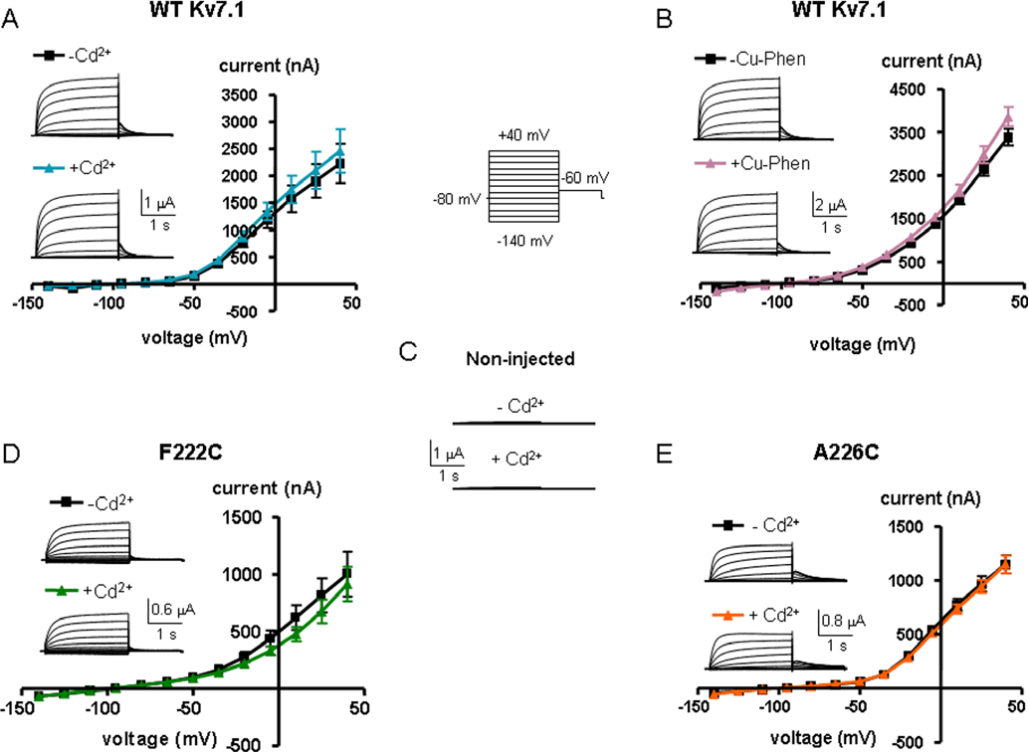
Effects of external Cd^2+^ or Cu-Phen on WT Kv7.1 (A and B), non-injected oocytes (C) and mutants F222C (D) and A226C (E). Oocytes were bathed in ND96 in the absence and presence of either 100 µM CdCl_2_ or 100 µM Cu-Phen. Currents were evoked by depolarizing steps from −140 mV to +40 mV in 15 mV increments (holding potential, −80 mV; tail potentials −60 mV), as shown in the scheme protocol between panels A and B. Shown are representative traces and current-voltage relations were determined as indicated.

The wild-type (WT) Kv7.1 subunit has nine endogenous cysteines, of which three are in principle accessible from the external solution and could thus potentially form metal or disulfide bridges (C136, C214 and C331, located in the S1, S3 and S6 transmembrane segments, respectively) (Figure 1). The remaining six cysteines are intracellularly located and likely inaccessible from the external solution; thus, they were not considered as potential coordinating residues. We first introduced single cysteine mutations into the short S3–S4 linker and the S4 N-terminus (residues 220–228) in the background of WT Kv7.1 channels and expressed the mutant channels in *Xenopus* oocytes (Figure 1). We examined the propensity of engineered cysteines to form metal or disulfide bridges, using extracellular Cd^2+^ ions (100 µM) or copper-phenanthroline (100 µM, Cu-Phen; 1∶3 ratio) respectively, and studied their effects on K^+^ currents by two-electrode voltage-clamp. As shown in Figure 2A and B, neither Cd^2+^ nor Cu-Phen significantly affected WT Kv7.1 channels. Current amplitudes, gating parameters and kinetics were unaffected by either reagents. This suggests that none of the endogenous cysteines in Kv7.1 is able to form a metal or disulfide bridge under these experimental conditions or alternatively, if any bridge is formed it does not affect channel function. The reducing agent dithiothreitol (DTT, 2 mM) had no effect on WT Kv7.1 (not shown). In non-injected *Xenopus* oocytes, none of the above reagents affected the endogenous oocyte currents (Figure 2C).

**Figure 3:**
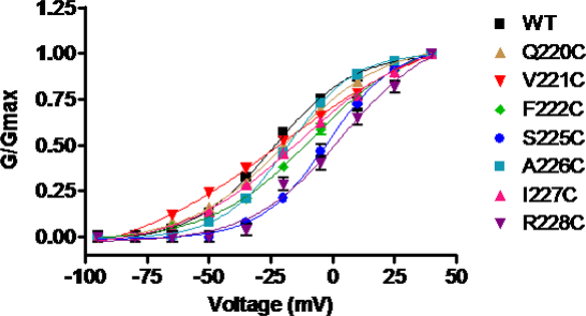
Conductance-voltage relations of WT Kv7.1 and mutants Q220C, V221C, F222C, S225C, A226C, I227C and R228C. Curves were fitted to one Boltzmann function. The following values were obtained: V_50_ = −24.1±2.3 mV, s = 16.3±2.1 mV (WT); V_50_ = −18.7±1.5 mV, s = 19.9±2.3 mV (Q220C); V_50_ = −20.5±3.5 mV, s = 33.8±7.1 mV (V221C); V_50_ = −8.3±1.3 mV, s = 20.5±2.2 mV (F222C); V_50_ = −1.9±1.5 mV, s = 13.8±1.7 mV (S225C); V_50_ = −18.0±1.1 mV, s = 13.4±1.0 mV (A226C); V_50_ = −13.6±1.7 mV, s = 23.7±3.2 mV (I227C); V_50_ = +3.9±4.1 mV, s = 18.5±3.5 mV (R228C). n = 6–20.

Among the cysteine mutants that were engineered (residues 220–228), A223C and T224C did not produce functional channels in *Xenopus* oocytes. While Q220C, V221C, A226C exhibited a similar voltage dependence of activation as WT Kv7.1 (V_50_ = −24.1±2.3 mV, n = 12), F222C, S225C and R228C displayed a significant right shift in their activation curve (Figure 3 and Table 1; V_50_ = −8.3±1.3 mV, V_50_ = −1.9±1.5 mV and V_50_ = +3.9±1.3 mV, respectively; n = 8–17, p<0.01). Neither Cu-Phen (100 µM) nor Cd^2+^ (100 µM) produced a significant effect on Q220C, V221C, F222C and A226C mutants as exemplified in Figure 2D and E.

**Table 1:** Electrophysiological parameters of WT and mutants Kv7.1 channels

Kv7.1 construct	V_50_ (mV)	I_inst_/I_max_ ratio		Rectification index	
		−Cd^2+^	+Cd^2+^	−Cd^2+^	+Cd^2+^
WT (12)	−24.1±2.3	0.11±0.03	0.10±001	0.035	0.022
Q220C (20)	−18.7±0.8	0.13±0.01	0.12±0.01	0.139	0.132
V221C (5)	−20.5±3.5	0.12±0.02	0.11±0.02	0.033	0.032
F222C (17)	−8.3±1.3 **	0.13±0.02	0.11±0.01	0.146	0.200
S225C (8)	−1.9±1.5 **	0.20±0.02	0.45±0.04 #	0.139	0.066
A226C (8)	−18.0±0.4	0.10±0.02	0.12±0.02	0.077	0.105
I227C (11)	−13.6±1.7 **	0.10±0.01	0.99±0.01 ##	0.064	0.481 ##
R228C (9)	+3.9±1.3 **	0.22±0.03	0.73±0.03 ##	0.125	0.536 ##
S225C-C136A (10)	−8.2±2.3 **	0.18±002	0.21±0.02	0.045	0.046
S225C-C214A (6)	−1.7±2.2 **	0.20±0.01	0.44±0.03 #	0.120	0.218
S225C-C331A (6)	−4.5±0.8 **	0.21±0.02	0.45±0.04 #	0	0
I227C-C136A (12)	−38.5±3.1 **	0.11±0.01	0.98±0.02 ##	0.026	0.506 ##
I227C-C214A (12)	−34.2±3.9 **	0.12±0.02	0.88±0.03 ##	0.071	0.722 ##
I227C-C331A (8)	−34.1±0.8 *	0.10±0.02	0.90±0.03 ##	0.014	0.254 ##
R228C-C136A (7)	+12.1±2.0 **	0.21±0.02	0.23±0.02	0.241	0.223
R228C-C214A (7)	+14.0±2.0 **	0.23±0.02	0.71±0.04 ##	0.218	0.624 ##
R228C-C331A (7)	+8.2±2.5 **	0.22±0.02	0.75±0.04 ##	0.126	0.558 ##

Activation curves were fit to a Boltzmann distribution, G/Gmax = 1/{1+exp[(V50-V)/s]}, where V_50_ is the voltage at which the current is half-activated and s is the slope factor. V_50_s were determined in the absence of Cd^2+^. Data are expressed as mean±SEM and in parentheses are indicated the number of cells.

* , p<0.05

** , p<0.01 vs. WT, (one way ANOVA followed by Dunnett's Multiple Comparison Test). To determine the I_inst_/I_max_ ratio and the rectification index, oocytes were recorded in the absence (−Cd^2+^) or presence of 100 µM Cd^2+^ (+Cd^2+^). The I_inst_/I_max_ ratio has been calculated by the ratio of the instantaneous current at the beginning of the pulse at +40 mV (Iinst) that follows the capacitive transient and the current at the end of the pulse at +40 mV (Imax). The larger is this ratio, the larger is the instantaneous open component.

# , p<0.05,

## , p<0.01 +Cd^2+^ versus −Cd^2+^ (two-tailed, Student's paired t test). The rectification index corresponds to the ratio of the current amplitude measured at −140 mV to that measured at −5 mV. The larger is this ratio, the stronger is the constitutive open leak K^+^ current component. Due to lack of space, are shown only the means (without SEMs) of the rectification index for the same number of tested cells indicated in parentheses.

### Mutant I227C is stabilized in the open state by Cd^2+^ and Cu-phen

**Figure 4:**
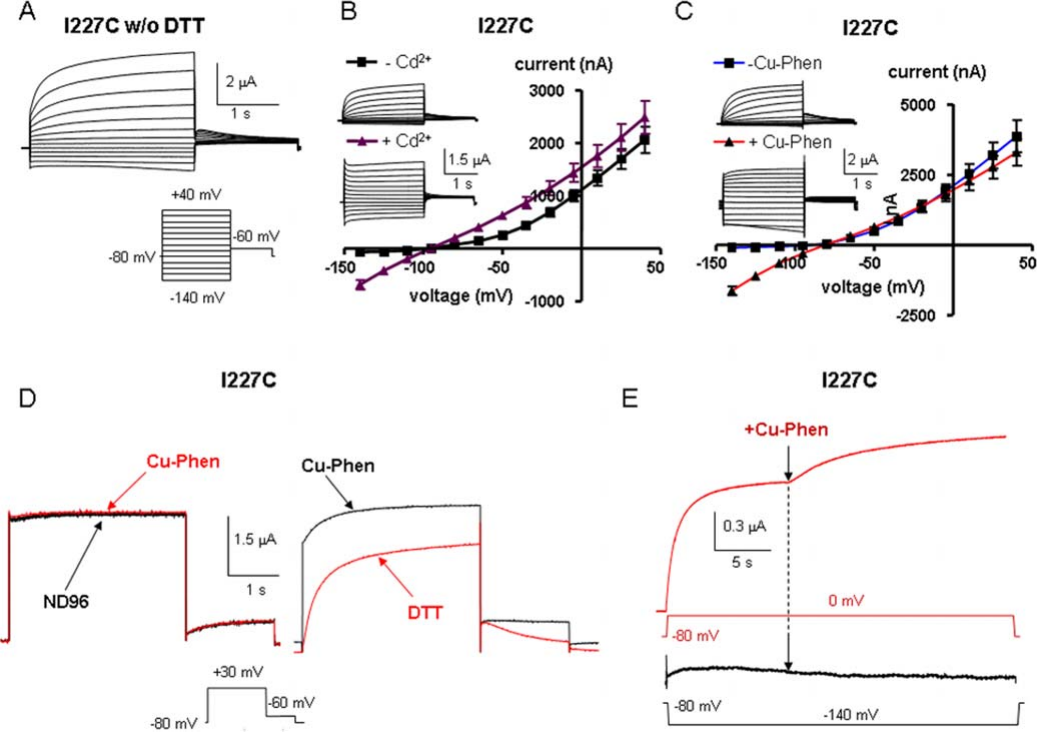
Effects of external Cd^2+^ and Cu-Phen on mutant I227C. (A) Representative traces recorded from an oocyte expressing I227C and bathed in ND96 without (w/o) prior incubation with 100 µM DTT (1 hour at 20°C) and without Cd^2+^ or Cu-Phen; Currents were evoked as in Figure 2. (B) Oocytes expressing I227C channels were first pre-incubated with 100 µM DTT (1 hour at 20°C) and subsequently washed with ND96 in the absence or presence of 100 µM CdCl_2_. (C) Same as in B but with 100 µM Cu-Phen. Shown are representative traces and current-voltage relations that were determined as indicated. (D) Representative traces of an oocyte expressing mutant I227C, bathed in ND96 containing 100 µM Cu-Phen and subsequently washed with either ND96 (left panel) or ND96 containing 1 mM DTT (right panel). Currents were evoked by a step depolarization from −80 mV to +30 mV. Similar results have been obtained in 6 other cells. (E) Representative traces of an oocyte expressing I227C, preincubated for 10 min with 100 µM Cu-Phen at −80 mV and then washed out for 5 min with ND96 at −80 mV. Currents were then evoked either by a depolarizing step to 0 mV (red trace) or by a hyperpolarizing step to −140 mV (black trace), after which a fast re-application of Cu-Phen was applied (red or black trace after the arrow). Similar results have been obtained in 5 other cells.

When recording of mutant I227C was performed without prior incubation of oocytes with 100 µM DTT (1 hour at 20°C under no voltage-clamp), large K^+^ currents were observed with a characteristic instantaneous rising component (at +40 mV, I_inst_/I_max_ ratio = 0.40±0.05; n = 11) and a partial loss of the voltage dependence. This latter feature was reflected by significant inward K^+^ currents evoked upon hyperpolarization (Figure 4A). Sometimes, tonic currents were recorded with a linear ohmic K^+^ current-voltage relation (Erev = −94±2 mV, n = 11). Following one hour preincubation of oocytes with 100 µM DTT, these features disappeared (at +40 mV, I_inst_/I_max_ ratio = 0.1±0.01; n = 11, p<0.01) and I227C channels regained the properties of clear voltage dependence and outward-rectification (Figure 4B and C, upper inset). These observations suggest that in I227C channels, disulfide bonds form spontaneously and stabilize the mutant channels in the open state. Therefore, to prevent spontaneous openings and to check the effects of Cd^2+^ and Cu-Phen, we routinely preincubated the oocytes with 100 µM DTT. Removal of DTT by external application of 100 µM Cd^2+^ to oocytes expressing I227C channels, rapidly converted the slow voltage-dependent activation into instantaneous leak K^+^ current (Figure 4B, lower inset; τact slow = 981±138 ms vs. τact fast = 90±10 ms, at +40 mV, n = 10), with an increased I_inst_/I_max_ ratio (Table 1). This drastic change is reflected by the conversion of the outwardly-rectifying K^+^ current into a linear I-V relation (Fig. 4). Consequently, Cd^2+^ potently increased the amplitude of the inward current (by ∼11.8-fold at −140 mV, n = 11, p<0.01) while slightly enhancing that of the outward current (by ∼1.2-fold at +40 mV). A significant activation was already observed at 25 µM Cd^2+^ (∼5-fold increase at −140 mV, n = 6, p<0.01). To further quantify the Cd^2+^ effect, we calculated the rectification index which corresponds to the ratio of the current amplitude measured at −140 mV to that measured at −5 mV. The larger is this ratio, the stronger is the constitutive open leak K^+^ current component (Table 1). Thus, for I227C channels the rectification index is 0.064 and 0.481 in the absence and presence of 100 µM Cd^2+^, respectively. These effects of Cd^2+^ were substantially reversed upon a 5 min washout, with yet some trend of spontaneous openings. We reasoned that if the mutant I227C is able to form a metal bridge, which is likely involved in the activating effect of Cd^2+^, it should also be capable of forming a covalent disulfide bond induced by Cu-Phe, with the same functional consequences on channel activity. We obtained very similar results when oocytes expressing I227C were externally exposed to 100 µM Cu-Phen. Cu-Phen progressively converted the voltage-dependent outwardly-rectifying K^+^ current into an instantaneous leak K^+^ current (Figure 4C). Thus, Cu-Phen strongly stimulated the inward K^+^ current at negative potentials (by ∼20-fold at −140 mV; n = 10, p<0.001). The stimulation of I227C currents induced by Cu-Phen (including the positive holding current, Figure 4D, left) could not be reversed by washout using the ND96 buffer, but was fully reversible using ND96 containing 1 mM DTT, a reagent which reduces disulfide bridges into free sulfhydryl-cysteines (Figure 4D, right). Next, we examined the state-dependence of disulfide bridge formation. When oocytes are preincubated for 10 min with Cu-Phen (100 µM) and subsequently washed out for 5 min with ND96, all at −80 mV, opening the I227C channels by a step depolarization to 0 mV produced a time- and voltage-dependent K^+^ current (red trace before the arrow) without any instantaneous component (Figure 4E). In contrast, fast reapplication of Cu-Phen during depolarization to 0 mV gradually stimulated the current within second time-scale (Figure 4E, red trace after the arrow). Using an identical preincubation protocol of Cu-Phen (10 min preincubation and 5 min wash at −80 mV), a step hyperpolarization to −140 mV did not produce any inward K^+^ current (Figure 4E, lower panel, black trace before the arrow). Similarly, fast reapplication of Cu-Phen during hyperpolarization to −140 mV did not generate inward currents (black trace after the arrow). Altogether, these data suggest an open state-dependence for disulfide bridge formation in mutant I227C.

Knowing that Cd^2+^ should be coordinated by at least two electron-donor lewis bases such as cysteines and that one needs two cysteines to form a disulfide bridge using Cu-Phen, then potential contributions of the other cysteines in I227C mutant channels can be examined. There are two possibilities: (1) the metal or disulfide bridge formation arises from an interaction between the engineered cysteine at position 227 in S4 and one of the three externally accessible endogenous cysteines in Kv7.1, which are C136 in S1, C214 in S3 and C331 in S6; or (2) the bridge formation arises from an interaction between the engineered cysteine at position 227 of two adjacent subunits.

**Figure 5:**
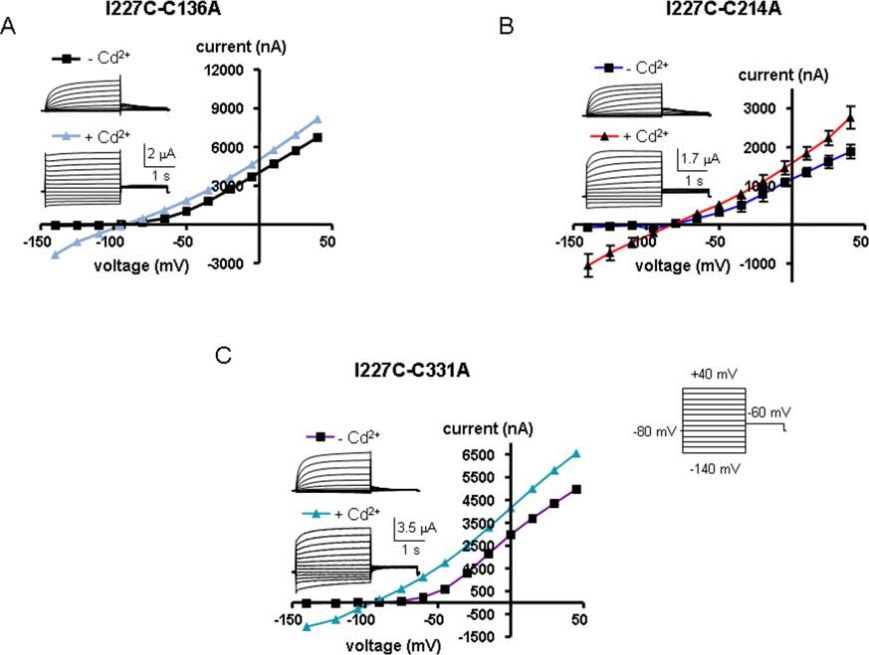
Effects of external Cd^2+^ on the double mutants I227C-C136A (A), I227C-C214A (B) and I227C-C331A (C). Oocytes were bathed in ND96 in the absence and presence of 100 µM CdCl_2_. Currents were evoked as in Figure 2. Shown are representative traces and current-voltage relations.

To determine which possibility prevails, we constructed double mutant channels where an alanine mutation was introduced in place of one of the three accessible endogenous cysteines in the background of the I227C mutant. Thus, we checked the impact of external Cd^2+^ on the double mutants I227C-C136A, I227C-C214A and I227C-C331A (Figure 5). Though activated at more negative potentials, the three double mutants exhibited similar characteristics of voltage-dependent outward-rectifying K^+^ channels when compared to I227C (Table 1 and Figure 5, traces under -Cd^2+^). As shown in Figure 5, external application of 100 µM Cd^2+^ switched the slowly activating voltage-dependent conductance into an instantaneous linear leak K^+^ current, and none of the three double mutants was able to suppress or even reduce the effect of Cd^2+^. Like I227C, the three double mutants exhibited a high I_inst_/I_max_ ratio and rectification index, reflecting a strong constitutive leak K^+^ current component (Table 1). These results suggest that, upon depolarization, a disulfide bridge could form between two I227C of two adjacent VSDs, a process that stabilizes the channel open state (see model and discussion).

### Mutant R228C is stabilized in the open state by Cd^2+^ and Cu-phen

**Figure 6:**
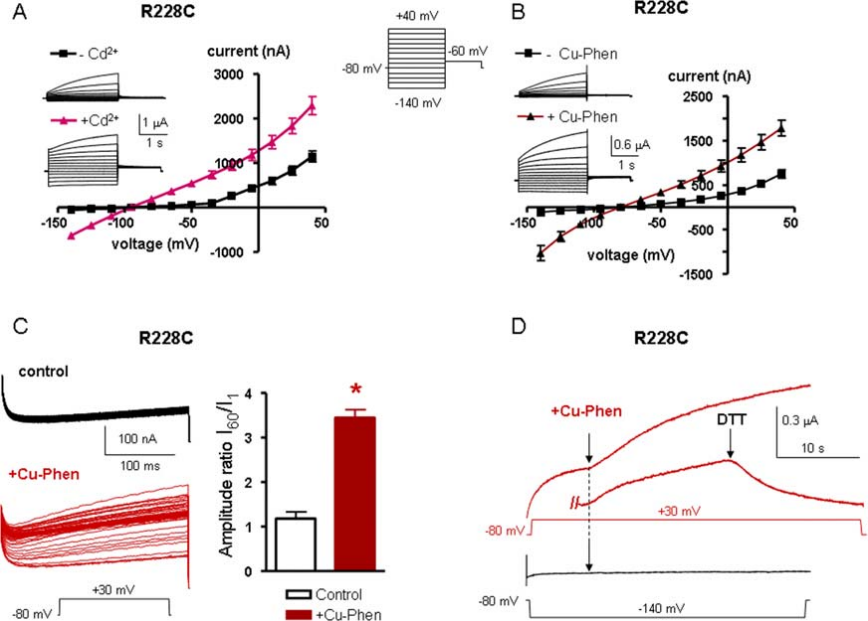
Effects of external Cd^2+^ and Cu-Phen on mutant R228C. Oocytes were bathed in ND96 in the absence and presence of 100 µM CdCl_2_ (A) or 100 µM Cu-Phen (B). Shown are representative traces and current-voltage relations were determined as indicated. (C) R228C channels were opened by depolarization to +30 mV in a train of 60 sweeps at 0.2 Hz, in the absence (black traces) or presence of 100 µM Cu-Phen (red traces). The increase in current was calculated by the ratio of the amplitude of the 60^th^ sweep to that of the 1^st^ sweep. (D) Representative traces of an oocyte expressing R228C, preincubated for 10 min with 100 µM Cu-Phen at −80 mV and then washed out for 5 min with ND96 at −80 mV. Currents were evoked either by a depolarizing step to +30 mV (red trace) or by a hyperpolarizing step to −140 mV (black trace), after which a fast reapplication of Cu-Phen was applied (red or black trace after the arrow). Also shown, is the reversal by DTT of the current increase produced by the fast reapplication of Cu-Phen at +30 mV. Similar results have been obtained in 5 other cells.

**Figure 7:**
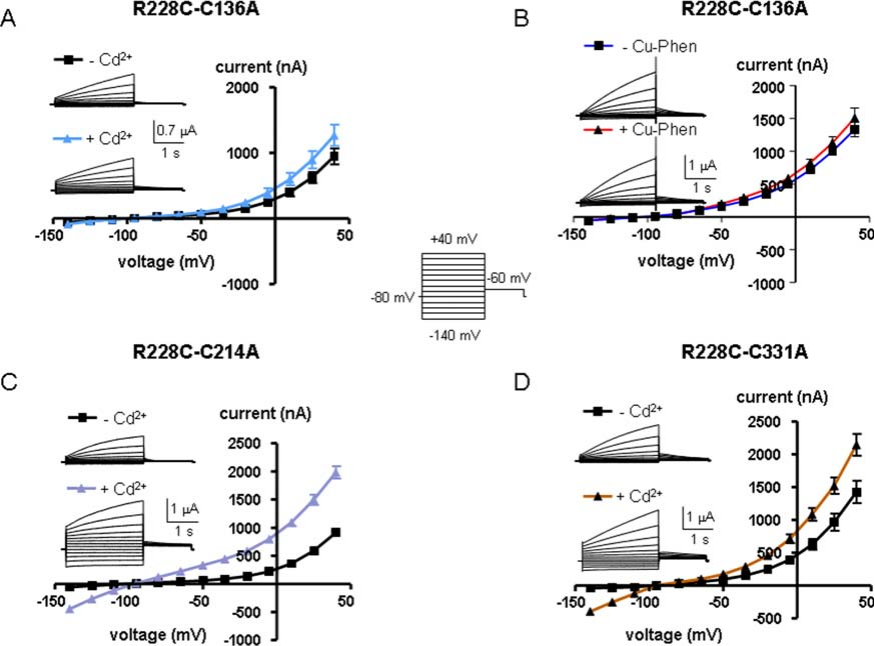
Effects of external Cd^2+^ (A) or Cu-Phen (B) on the double mutant R228C-C136A and of Cd^2+^ on R228C-C214A (C) and R228C-C331A (D). Oocytes were bathed in ND96 in the absence and presence of 100 µM CdCl_2_ or 100 µM Cu-Phen. Shown are representative traces and current-voltage relations were determined as indicated.

In this mutant, the cysteine has been engineered at the first arginine of S4, producing a slowly activating K^+^ current when compared to WT Kv7.1, which is reminiscent of the slow *I_KS_* activation kinetics (at +40 mV, τ_act_ = 1,509±60 ms for R228C, compared to τ_act_ fast = 80±3 ms and τ_act_ slow = 736±44 ms for WT Kv7.1, n = 8; p<0.01) (Figure 6). As mentioned above, mutant R228C is also right-shifted in its voltage dependence of activation (by ∼+28 mV). Like for mutant I227C, we routinely preincubated the oocytes with 100 µM DTT to keep the mutant channels in the closed state. Following removal of DTT and exposure of R228C-expressing oocytes to 100 µM Cd^2+^ or to 100 µM Cu-Phen, the slowly activating *I_KS_*-like K^+^ current demonstrated a large instantaneous component with a significant increase of the I_inst_/I_max_ ratio (Figure 6A and B and Table 1). In addition, the outwardly-rectifying shape of the R228C current-voltage relation (I-V) switched to a nearly linear leak character with a higher rectification index, reflecting a powerful stimulation of the inward current (by ∼14-fold and 16-fold for Cd^2+^ and Cu-Phen, respectively; n = 9–18, p<0.001) and a significant enhancement of the outward current (by ∼2-fold and 2.4-fold for Cd^2+^ and Cu-Phen, respectively; n = 9–18, p<0.001) (Figure 6A and B and Table 1). While the effects of Cd^2+^ were reversed upon extensive washout with ND96, the effects of Cu-Phen could be reversed only in the presence of 1 mM DTT (see Figure 6D). Figure 6C and D illustrate the state-dependence of disulfide bridge formation in R228C by Cu-Phen. When R228C channels were opened by a train stimulus (60 sweeps to +30 mV for 250 ms at 0.2 Hz), the slowly developing current did not increase significantly during the repetitive stimulation (by ∼1.2-fold). However, when the same train protocol was performed in the presence of 100 µM Cu-Phen, the current markedly increased (by 3.45-fold; n = 6, p<0.01) and progressively developed an instantaneous component (Figure 6C). This data suggests that disulfide bridge formation by Cu-Phen produced an accumulation of channel open-state. Like for mutant I227C, using a preincubation protocol with Cu-Phe (10 min preincubation and 5 min wash at −80 mV), we found that opening the R228C channels by a step depolarization to +30 mV produced a slowly developing time- and voltage-dependent K^+^ current (red trace before the arrow) without any instantaneous component. In contrast, fast reapplication of Cu-Phen during depolarization to +30 mV markedly enhanced the current (Figure 6D, red trace after the arrow), a feature which was reversed following exposure to 1 mM DTT. Following 10 min preincubation with Cu-Phen and 5 min wash at −80 mV, a step hyperpolarization to −140 mV did not generate any inward K^+^ current (Figure 6D, lower panel, black trace before the arrow). Fast reapplication of Cu-Phen during hyperpolarization to −140 mV did not induce inward currents (black trace after the arrow). Similar to I227C, these data suggest an open state-dependence for disulfide bridge formation in mutant R228C. Then, we checked the impact of external Cd^2+^ and Cu-Phen on the double mutants R228C-C136A, R228C-C214A and R228C-C331A, where an alanine mutation was introduced in place of one of the three accessible endogenous cysteines in the background of the R228C mutation (Figure 7). In the absence of Cd^2+^ or Cu-Phen, the three double mutants exhibited gating characteristics similar to those of R228C. Figure 7C and D shows that external application of 100 µM Cd^2+^ to R228C-C214A and R228C-C331A mutants switched their slow outwardly-rectifying voltage-dependent conductance into currents which display: (i) large instantaneous outward and inward components and (ii) I-V patterns approximating a linear K^+^ leak with high rectification index (Table 1). Similar results were obtained with Cu-Phen (not shown). In contrast, the double mutant R228C-C136A totally suppressed the ability of Cd^2+^ and of Cu-Phen to activate the current both in the outward and inward direction and to induce an instantaneous component (figure 7A and B and Table 1). Thus, the current pattern of R228C-C136A in the presence of Cd^2+^ or Cu-Phen is similar to that of R228C obtained in the absence of Cd^2+^ or Cu-Phen; i.e., time- and voltage-dependent as well as outwardly-rectifying. This data suggests that following depolarization, a disulfide bridge could form between R228C in S4 and C136 in S1, which stabilizes the channel open state (see model and discussion).

### Mutant S225C is stabilized in the closed state by Cd^2+^ and Cu-phen

**Figure 8:**
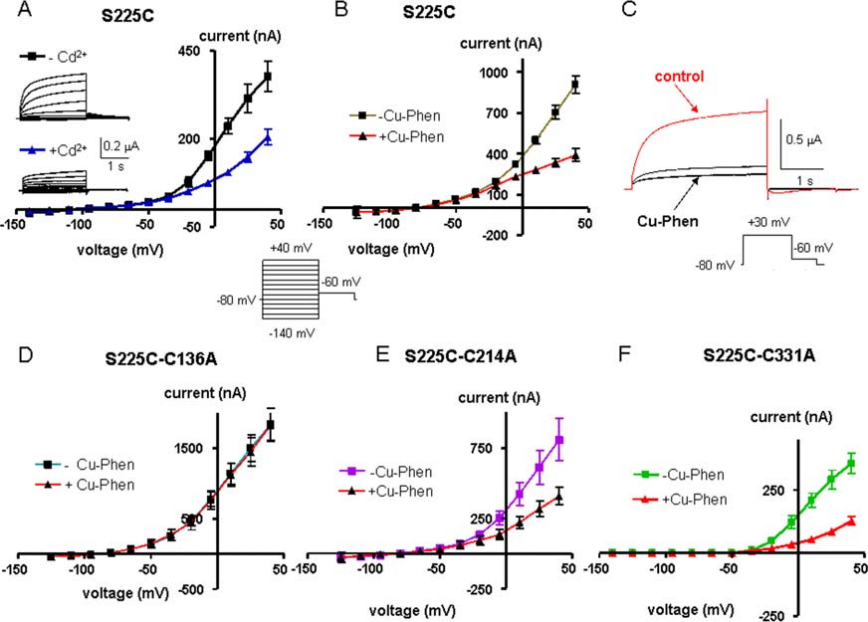
Effects of external Cd^2+^ (A) or Cu-Phen (B) on mutant S225C. Oocytes were bathed in ND96 in the absence and presence of 100 µM CdCl_2_ or 100 µM Cu-Phen. Shown are representative traces and current-voltage relations were determined as indicated. (C) Shown are representative traces of an oocyte expressing mutant S225C before (red trace) and after (black trace) 10 min preincubation with Cu-Phen (100 µM) at −80 mV plus 5 min wash at −80 mV with ND96. Currents were evoked by a step depolarization to +30 mV. Similar results have been obtained in 6 other cells. Effects of Cu-Phen on the double mutants S225C-C136A (D), S225C-C214A (E) and S225C-C331A (F). Currents were evoked as in Figure 2. Current-voltage relations were determined as indicated.

Mutant S225C produced a current amplitude lower than that of WT Kv7.1 (at +40 mV, ∼3-fold lower than WT Kv7.1; Figure 8), with a right-shift of the activation curve (V_50_ = −1.9±1.5 mV, n = 8). In contrast to I227C or R228C, when mutant S225C was treated with 100 µM Cd^2+^ or 100 µM Cu-Phen a marked decrease of the current amplitude was observed with a 46% and 57% decrease, respectively, at +40 mV (Figure 8A and B). The decrease of S225C currents by Cu-Phen could not be reversed upon washout, unless using ND96-containing DTT (not shown). When Cu-Phen (100 µM) was preincubated at −80 mV for 10 min and then washed out for 5 min, opening of S225C channels by a step depolarization to +30 mV was markedly inhibited (black traces), as compared to the same oocyte before treatment with Cu-Phen (red trace, control) (Figure 8C). This result suggests that disulfide bridge formation in S225C occurs in the closed state. Notably, the double mutants S225C-C214A and S225C-C331A generated currents that were highly sensitive to inhibition by Cu-Phen (at +40 mV, 50% and 64% inhibition, respectively; Figure 8E and F). In contrast, mutant S225C-C136A was totally insensitive to inhibition by Cu-Phen (Figure 8D). Altogether, the data suggest that a disulfide bridge could form between S225C in S4 and C136 in S1, in the channel closed state (see model and discussion).

## Discussion

Numerous efforts have been invested to elucidate the nature of the VSD's motion using *Shaker* channels as a template but yet the gating mechanisms remain controversial mainly because the structure of the closed state is unknown [8]. A few functional studies have addressed this key question in other Kv channels to generalize their gating principles [9]. In particular, no study is available for Kv7.1 channels which assemble with KCNE1 to produce the slow *I_KS_* current, a major repolarizing K^+^ conductance of the cardiac action potential [10]. In this study, a cysteine scan of Kv7.1 S4 and structural modeling constraints suggest a key role of the S1 segment in steering S4 motions and interactions during the gating process.

**Figure 9:**
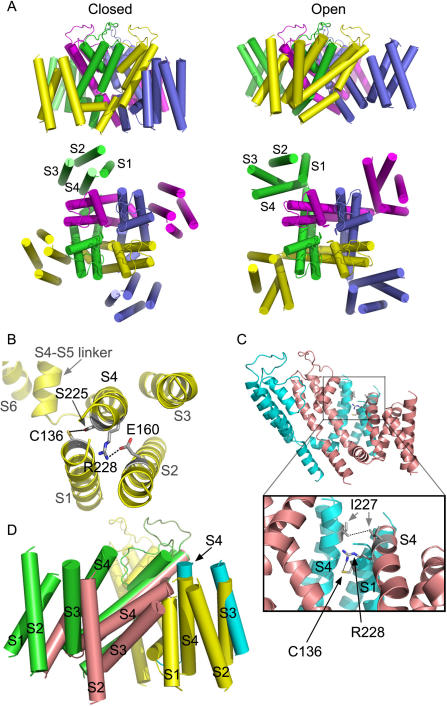
Plausible structural models accounting for the gating conformations of the voltage sensor domains of Kv7.1. (A) The closed (left) and open (right) conformations of Kv7.1 in side view (upper part) or top view (lower part). Each subunit is colored differently while alpha helices are shown as cylinders. (B) Top view of a subunit that reflects the closed state, where R228 becomes close to Glu160 (2.7 Å) and S255 to Cys136 (5.1 Å) within the same subunit. The indicated side chains are colored in CPK (grey, blue, red and yellow colors correspond to carbon, nitrogen, oxygen and sulfur atoms, respectively). (C) Side view of two adjacent subunits, which represent an intermediate open state (salmon) and an intermediate closed state (cyan). The two subunits are viewed from the inner part of the pore in horizontal outward direction. The inset shows the distance between Ile227 of two neighboring VSDs (5.2 Å) as well as the distance between R228 and Cys136 of the adjacent VSD (4.6 Å). (D) Adjacent VSDs in the open state (green), the intermediate open state (salmon), the intermediate closed state (cyan) and the closed state (yellow). Note that the VSD of the intermediate open state (salmon) tilts towards the neighboring VSD to interact with the S4 of the intermediate closed state (cyan), which undergoes axial rotation and translation from the closed state (yellow).

In the absence of KCNE1, we found that, in the channel closed state disulfide bridge could form between S225C in S4 and C136 in S1. In the channel open state, our results identify two major constraints which include an interaction between two I227 of two adjacent S4 segments as well as between C136 in S1 and R228 in S4 of two neighboring VSDs (see model below). We showed that disulfide and metal bridges can form in homomeric I227C channels only upon depolarization, which locks the channels in the open state. Notably, none of the three externally accessible cysteines of Kv7.1 are coordinating ligand partners for I227C as the three double mutants I227C-C136A, I227C-C214A and I227C-C331A were unable to suppress or even reduce the activating effect of Cd^2+^. The most plausible interpretation of these data is that the bridge formation arises from the interaction between engineered I227C from two adjacent subunits. This data for Kv7.1 is reminiscent of a previous work performed in *Shaker* channels showing that cysteines introduced at position L361 of S4 (equivalent to I227 in Kv7.1) can be crosslinked by an intersubunit disulfide bridge [11], [12]. Our results reveal an additional open state constraint, reflected by the depolarization-dependent formation of inter-subunit disulfide and metal bridges between R228C in S4 and C136 in S1, thus locking the channels in the open state. These experimental data guided us to build a structural model, using the Kv1.2 crystal structure as a template [13]. We imposed closing of the channel by: (i) moving each VSD together with the S4–S5 linker and the lower part of S6 (see methods), and (ii) bringing S225 in S4 near to C136 in S1 within the same VSD (5.1 Å, between oxygen and sulfur atoms, center to center). As such, when the channel moves from the open to the closed state, the VSDs slightly move laterally and downward as shown in Figure 9A. Since the extracellular S3–S4 loop comprises only three amino acids, S3 was moved together and around S4, preserving their upper parts close to each other. Both S3 and S4 could move relatively independently from S1 and S2, owing to a 20-amino acid long loop sequence residing between the bottom parts of S2 and S3 (see model building in Methods). The open to closed state transition requires a counterclockwise rotation (∼190°) and a downward translation of S4 (full translation is ∼12 Å). This leads residues E160 in S2 (E283 in *Shaker*) and R228 in S4 (R362 in *Shaker*) to be within atomic proximity (2.7 Å), indicating ion pairing (Figure 9B). This latter assignment is consistent with previous experimental studies showing that in *Shaker* channels, E283 at S2 packs against R362 at S4 [14]. The closed state constraint of C136 in S1 is appealing when considering a recent work performed in *Shaker* channels which showed that a histidine mutant of I241 (equivalent of C136 in Kv7.1) generates inward currents at hyperpolarized potentials (closed state), suggesting that it forms part of a hydrophobic plug that splits the water-accessible crevices [15]. At hyperpolarized potentials, it was also found that I241C (C136 in Kv7.1) can spontaneously form disulfide and metal bridges with R362C (R228 in Kv7.1). Consistent with these data in *Shaker*, our closed state model also predicts that R228 (S4) points close to C136 (S1) (Figure 9B). These features are in line with a recent study predicting that in the closed state (down state), S4 contacts S5 along one helical face and S1 on its N-terminal half of the opposite face [16]. To operate the closed to open transition, the results and the modeling work suggest a cooperative interaction between adjacent VSDs, where S4 from the activated VSD undergoes a clockwise axial rotation (∼190°) and an outward translation (∼12 Å). This S4 motion is accompanied by a lateral and upward movement of the entire VSD and a rocking movement toward the extracellular tip of the S4 of the resting adjacent subunit (Figure 9C and D). This rocking motion allows I227 which resides one helical turn below the tip of the activated S4 to get close to an adjacent I227 of the neighboring resting S4 when the latter starts to rotate (by ∼90°) and move upward (by ∼4 Å) (Figure 9C). At this ‘intermediate’ stage, the distance between the two I227 of the neighboring VSDs is 5.2 Å (between the two Cδ atoms, center to center). Such proximity would enable disulfide bridging if cysteine is substituted at position 227. Interestingly, this movement also enables R228 of the activated VSD to come to atomic proximity with C136 of S1 of the adjacent resting VSD (4.6 Å between NH1 and sulfur atoms, center to center, respectively) (Figure 9C).

Gating of Kv channels is thought to involve two kinds of VSD transmembrane motions: an early independent movement of the four VSDs from a resting to an activated conformation, followed by a concerted VSD motion underlying the final opening transition [17]–[19]. The inter-VSD interactions between two I227 of adjacent S4s and between R228 in S4 and C136 in S1 of adjacent VSDs provide a ground for cooperative interactions between subunits. Although our model predicts a different VSD trajectory for channel gating, the notion of inter-VSD interaction described here is in agreement with a previous work in *Shaker* channels where the extracellular tip of an activated S4 was suggested to be in close proximity to the extracellular end of the resting S4 in the adjacent subunit [11], [12]. Compared to other gating models [4], [7], [8], [15], [16], [20], [21], our model for the closed to open transition in Kv7.1 can be envisioned as a combination of two previously described mechanisms with intra- and inter-VSD motions (Figure 9A and D). While the clockwise axial rotation and outward translation of S4 is consistent with the ‘helical screw model’ [8], [20], the lateral and upward motion of the entire VSD is reminiscent of the ‘paddle model’ [7]. In addition, our model predicts a rocking motion of an activated VSD onto an adjacent resting VSD, which may account for Kv channel cooperative activation (Figure 9D). The model is also consistent with recently described closed state topology of S4 with respect to S1 (down state) [15], [16], [20], [21], where replacement of the first arginine R362 in *Shaker* (R228 in Kv7.1) by a histidine or by a small uncharged amino acid generates a proton or a cation (omega current) pore, respectively [4], [14]. Interestingly, a recent high resolution structure of the paddle of a Kv2.1-1.2 chimera channel in a membrane-like environment reveals that negatively charged amino acids in the VSD are grouped into two clusters: an external and an internal negative clusters whose residues are highly conserved in Kv7.1 channels [22]. While the external negative cluster is located in an external aqueous cleft, the internal negative cluster belongs to a buried network of charged amino acids. Notably, a conserved phenylalanine (F167 in Kv7.1), positioned near the midpoint of the membrane, separates these external and internal negative clusters. In the open conformation, the R4 α carbon (R237 in Kv7.1) is expected to be located at the level of this phenylalanine (F167 in Kv7.1). Thus, it was suggested that in the channel open state a histidine side chain at this R4 location is well positioned to flip above and below the conserved phenylalanine side chain by rapid rotamer exchange, allowing protons to be transferred [22]. It remains to determine whether such proton currents could be measured in Kv7.1 channels under these conditions. Overall, our data indicate a crucial role of S1 in funneling S4 motion during Kv7.1 gating. Furthermore, the large VSD motion that we infer for Kv7.1 channel gating is in line with the most recent structural and functional studies [22], [23], indicating that the entire voltage sensing domain moves in a relatively unconstrained environment within the lipid membrane.

## Materials and Methods

### Channel expression into Xenopus oocytes

Female *Xenopus* Laevis frogs were purchased from Xenopus 1 (Dexter, Michigan, USA). The procedures followed for surgery and maintenance of frogs were approved by the animal research ethics committee of Tel Aviv University and in accordance with the Guide for the Care and Use of Laboratory Animals (1996. National Academy of Sciences, Washington D.C.). Frogs were anaesthetized with 0.15% tricaine (Sigma). Pieces of the ovary were surgically removed and digested with 1 mg/ml collagenase (type IA, Sigma) in Ca^2+^-free ND96 for about one hour, to remove follicular cells. Stage V and VI oocytes were selected for cRNA injection and maintained at 18°C in ND96 (in mM: 96 NaCl, 2 KCl, 1.8 mM CaCl_2_, 1 MgCl2 and 5 HEPES titrated to pH = 7.5 with NaOH), supplemented with 1 mM pyruvate and 50 µg/ml gentamycin. The human Kv7.1 cDNA (in pGEM vector) was linearized by Not1. This vector served also as a template to generate the Kv7.1 mutants, using site-directed mutagenesis performed by the QuikChange (Stratagene) method. All mutant sequences were verified by DNA sequencing. Capped complementary RNA was transcribed by the T7 RNA polymerase, using the mMessage mMachine transcription kit (Ambion Corp). The cRNA size and integrity was confirmed by formaldehyde-agarose gel electrophoresis. Expression of WT and Kv7.1 mutants was performed by injecting 40 nl per oocyte (5 ng cRNA) using a Nanoject injector (Drummond, USA).

### Electrophysiology

Electrophysiological recording were performed as previously described [24]. Briefly, standard two-electrode voltage-clamp measurements were performed at room temperature (22°C–24°C) 2–5 days following cRNA microinjection. Oocytes were placed into a 100 µl recording chamber and superfused with a modified ND96 solution (containing 0.1 mM CaCl_2_) using a fast perfusion system which operates under controlled N_2_ pressure allowing constant perfusion velocity of 3.9–4.2 ml/min. The exchange of solutions was performed by computer-controlled pinch valves (ALA-VM8, ALA Scientific Instruments). A home made manifold having virtually no void volume and very narrow connecting tubes prevented backward flow upon valve switch. The bath solution was completely replaced within 1.5 seconds, allowing a solution exchange time of about 25 ms around the oocyte. Whole-cell currents were recorded using a GeneClamp 500 amplifier (Axon Instruments). Stimulation of the preparation, and data acquisition were performed using the pCLAMP 6.02 software (Axon Instruments) and a 586 personal computer interfaced with a Digidata 1200 interface (Axon Instruments). Glass microelectrodes (A-M systems, Inc) were filled with 3M KCl and had tip resistances of 0.2–0.5 MΩ. Current signals were digitized at 1 kHz and low pass filtered at 0.2 kHz. Errors introduced by the series resistance of the oocytes were not corrected and were minimized by keeping expression of the currents below 10 µA.

### Data Analysis

Conductance (G) obtained from tail current amplitudes or from steady-state currents (when deactivation was very fast) was calculated by the following equation G = I/(V- Vrev) where the calculated reversal potential Vrev was −98±2 mV (n = 10).. G was then, normalized to the maximal conductance value, Gmax. Activation curves were fitted by a single Boltzmann distribution G/Gmax = 1/{1+exp[(V50-V)/s], where V_50_ is the voltage at which the current is half-activated and s is the slope factor. The Iinst/Imax ratio has been calculated by the ratio of the instantaneous current at the beginning of the pulse at +40 mV (Iinst) that follows the capacitive transient and the current at the end of the pulse at +40 mV (Imax). The larger is this ratio, the larger is the instantaneous open component. The rectification index was calculated as the ratio of the current amplitude measured at −140 mV to that measured at −5 mV. The larger is this ratio, the stronger is the constitutive open leak K+ current component. All data were expressed as mean±SEM. Statistically significant differences between paired groups were assessed by a two-tailed Student's t-test. Statistically significant differences between unpaired groups were assessed using one way ANOVA followed by Dunnett's Multiple Comparison Test.

### Model building

#### The open state

As a first step, the sequence of the pore domain (S5-pore helix-S6 segment) of the human Kv7.1 (KCNQ1) channel (^257^IHR…VQQ^357^; SWISS-PROT entry P51787) was submitted for searching a homologous template in the SWISS-MODEL repository, a database for theoretical protein models (http://swissmodel.expasy.org/SWISS-MODEL.html). The search for homologous sequences of known 3D structure scored the mammalian Kv1.2 potassium channel (PDB ID code 2A79) with the highest probability to match as a structural template. The Kv7.1 sequence ^257^IHR…VQQ^357^ was therefore aligned with the ^323^ASM…YHR^419^ sequence of Kv1.2 using the program T-COFFEE, implemented in SWISS-MODEL. (Figure S1), and the alignment was submitted to automated comparative protein modeling via the SWISS-PROT alignment interface, as performed by Gibor et al [25]. The root mean square difference (rmsd) between the Kv7.1 structural model and the template (Kv1.2) was 0.11 Å for 96 Cα atoms of the aligned amino acids (0.27 Å for 384 backbone atoms). Note that this superposition does not include amino acids 290–294 as they form an extra loop structure in the turret region.

The same procedure was used for each of the sequences ^120^TRPF-PHE^139^ (S1), ^153^THR-SER^177^ (S2), ^198^ILE-LYS^218^ (S3) and ^222^PHE-PHE^256^ (S4 and the S4–S5 linker) of Kv7.1, according to the alignments presented in Figure S1. The resulting modeled segments fitted perfectly in three dimensions to their homologous backbone atoms in Kv1.2 and therefore the C-terminus of the S4–S5 linker (PHE256) could readily be fused to the N-terminal amino acid of the pore domain (ILE257). Four identical subunit models were organized around the axis of K^+^ conduction by superposition of 188 backbone atoms of amino acids THR311-GLN357 onto the segment THR373-ARG419 of Kv1.2. Energy minimization of the tetrameric Kv7.1 model was performed with the GROMOS96. No clashes within the individual subunits or at the subunit interfaces have been observed.

#### The closed state

To obtain the closed state, we first aligned the Kv7.1 open-state model with KcsA (PDB ID code 1K4C) by superposing the selectivity filter and the pore helix of the two structures, as performed by Long et al [13]. Then, the VSD (S1, S2, S3, S4 and the S4–S5 linker) and the lower part of S6 (ALA344-GLN356) were moved together using the bent around His258 (i.e., between S5 and the S4–S5 linker) as a pivot, until the lower part of the Kv7.1 S6 (ALA344-GLN356) aligned three-dimensionally with its homologous segment in KcsA (THR107-GLN119) (52 backbone atoms giving RMS value of 0.39 Å). At this stage, the VSDs adopted an excessively low topology and therefore they were manually tilted and lifted while preserving interactions between S6 and the S4–S5 linker to keep the bottom-pore constriction, closed. The latter step takes into account previous inferences about gating charges of S4 that move through a narrow pathway between open internal and external vestibules [21], [26], [27]. The S4 was rotated around its longitudinal axis by ∼190 degrees and was translated downward by ∼12 Å so as to account for the experimental results showing that, in the closed state, Cys substituted at position 225 interacts with Cys136 of the same subunit. This rotation-translational motion allows the first arginine of S4 (Arg228) to pass near Cys136 of the same VSD and to become, at the end of the movement, sufficiently close to Glu160 (in S2) to enable ion pairing. Such proximities were shown to take place between the homologous positions in other voltage-dependent K^+^ channels [14]. The C-terminus of S3 and the N-terminus of S4 (i.e., their most extracellular parts) are separated by only three amino acids. Hence, S3 was moved together and around S4 keeping their upper parts close to each other. Both S3 and S4 could move relatively far from S1 and S2, owing to a 20-amino acid long loop sequence residing between the bottom parts of S2 and S3.

#### The intermediate state

A major constraint that guided us to introduce the conformational changes in order to obtain the intermediate state relates to the open-state stabilizing interactions between two neighboring VSDs. Of particular importance is the observation that position 227 of one VSD gets very close to position 227 of an adjacent VSD. This is feasible only if a VSD moves upward during activation to the intermediate state where it can tilt toward- and interacts with a neighboring VSD that is still down in its closed-state conformation (resting state). For that purpose, we have used the flexible loop between S4 and S4–S5 helical linker (244QGG) as a pivoting hinge. The S4 of the closed-state conformation was rotated clockwise by ∼90–100 degrees and translated upward by ∼4 Å. In this intermediate transition, the side chains of two Ile227 of neighboring VSDs get close to each other, while R228 of S4 of the upper-tilted activated VSD interacts with the Cys136 in S1 of the neighboring VSD, as shown in Figure 9C. Note that energy was minimized in the closed and intermediate state models and no clashes take place in these two models, as well.

## Supporting Information

Figure S1 (0.62 MB PDF)
