# Supplementary material for: S1 Constrains S4 in the Voltage Sensor Domain of Kv7.1 K+ Channels
Source: PLoS One. 2008 Apr 9;3(4):e1935. doi: 10.1371/journal.pone.0001935 (PMC2275789; doi:10.1371/journal.pone.0001935)
Supplement: Figure S1 — (0.62 MB PDF) [file pone.0001935.s001.pdf]

Supporting Information

Figure S1. Sequence alignments of human Kv7.1 (Swiss-Prot P51787), rat Kv1.2 (PDB ID 2A79, chain B) and *Streptomyces lividans* KcsA (PDB ID 1BL8). Multiple sequence alignment corresponding to S1, S2, S3, S4 and the S4-S5 linker was performed with ClustalW using all sequences provided in the web site http://vkcdb.biology.ualberta.ca/ for the Kv1, 2, 3, 4 and 7 families. The alignment corresponding to the pore domain (S5-Pore helix-S6 segment) was performed with the program T-COFFEE (see Materials and methods). Black background indicates identical amino acids. Grey background indicates similarities according to size, hydrophobicity, aromaticity, charge and polarity. The letter "h" denotes alpha helix in the 3-D structural models of Kv7.1.

```
                S1
                hhhhhhhhhhhhhhhhhhhh
Kv7.1   120     WKCFVYHFAVFLIVLVCLIF
Kv1.2   161     PARIIAIVSVMVILISIVSF

                S2
                hhhhhhhhhhhhhhhhhhhhhhhhh
Kv7.1   153     TGTLFWMEIVLVVFFGTEYVVRLWS
Kv1.2   219     TDPFFIVETLCIIWFSFEFLVRFFA

                S3
                hhhhhhhhhhhhhhhhhhhhh
Kv7.1   198     ISIIDLIVVVASMVVLCVGSK
Kv1.2   255     MNIIDIVAIIPYFITLGTELA

                                        S4-S5 linker
                S4
                 hhhhhhhhhhhhhhhhhhhhh   hhhhhhhhhh
Kv7.1   222     FATSAIRGIRFLQILRMLHVDRQGGTWRLLGSVVF
Kv1.2   288     MSLAILRVIRLVRVFRIFKLSRHSKGLQILGQTLK

                S5                                                Pore helix
                 h hhhhhhhhhhhhhhhhhhhhhhhhhhh                    hhhhhhhhhhhhhh
Kv7.1   257     -IHRQELITTLYIGFLGLIFSSYFVYLAEKDAVNESGRVEFGSYADALWWGVVTVT
Kv1.2   323     -ASMRELGLLIFFLFIGVILFSSAVYFAEADERDS----QFPSIPDAFWWAVVSMT
KcsA     23     ALHWRAAGAATVLLVIVLLAGSYLAVLAERGAPG----AQLITYPRALWWSVETAT

                           S6
                           hhhhhhhhhhhhhhhhhhh hhhhhhhhhhhhhhh
Kv7.1   312     TIGYGDKVPQTWVGKTIASCFSVFAISFFALPAGILGSGFALKVQQ  357
Kv1.2   374     TVGYGDMVPTTIGGKIVGSLCAIAGVLTIALPVPVIVSNFNYFYHR  419
KcsA     75     TVGYGDLYPVTLWGRCVAVVVMVAGITSFGLVTAALATWFVGREQ-  119
```
